# Structural and Functional Characterization of a Complex between the Acidic Transactivation Domain of EBNA2 and the Tfb1/p62 Subunit of TFIIH

**DOI:** 10.1371/journal.ppat.1004042

**Published:** 2014-03-27

**Authors:** Philippe R. Chabot, Luca Raiola, Mathieu Lussier-Price, Thomas Morse, Genevieve Arseneault, Jacques Archambault, James G. Omichinski

**Affiliations:** 1 Département de Biochimie et Médicine Moléculaire, Université de Montréal, Succursale Centre-Ville, Montréal, Québec, Canada; 2 Institut de Recherches Cliniques de Montréal, Montréal, Québec, Canada; Wistar Institute, United States of America

## Abstract

Infection with the Epstein-Barr virus (EBV) can lead to a number of human diseases including Hodgkin's and Burkitt's lymphomas. The development of these EBV-linked diseases is associated with the presence of nine viral latent proteins, including the nuclear antigen 2 (EBNA2). The EBNA2 protein plays a crucial role in EBV infection through its ability to activate transcription of both host and viral genes. As part of this function, EBNA2 associates with several host transcriptional regulatory proteins, including the Tfb1/p62 (yeast/human) subunit of the general transcription factor IIH (TFIIH) and the histone acetyltransferase CBP(CREB-binding protein)/p300, through interactions with its C-terminal transactivation domain (TAD). In this manuscript, we examine the interaction of the acidic TAD of EBNA2 (residues 431–487) with the Tfb1/p62 subunit of TFIIH and CBP/p300 using nuclear magnetic resonance (NMR) spectroscopy, isothermal titration calorimeter (ITC) and transactivation studies in yeast. NMR studies show that the TAD of EBNA2 binds to the pleckstrin homology (PH) domain of Tfb1 (Tfb1PH) and that residues 448–471 (EBNA2_448–471_) are necessary and sufficient for this interaction. NMR structural characterization of a Tfb1PH-EBNA2_448–471_ complex demonstrates that the intrinsically disordered TAD of EBNA2 forms a 9-residue α-helix in complex with Tfb1PH. Within this helix, three hydrophobic amino acids (Trp458, Ile461 and Phe462) make a series of important interactions with Tfb1PH and their importance is validated in ITC and transactivation studies using mutants of EBNA2. In addition, NMR studies indicate that the same region of EBNA2 is also required for binding to the KIX domain of CBP/p300. This study provides an atomic level description of interactions involving the TAD of EBNA2 with target host proteins. In addition, comparison of the Tfb1PH-EBNA2_448–471_ complex with structures of the TAD of p53 and VP16 bound to Tfb1PH highlights the versatility of intrinsically disordered acidic TADs in recognizing common target host proteins.

## Author Summary

Infection with the Epstein-Barr virus (EBV) is linked to a number of human diseases and the nuclear antigen EBNA2 is one of nine viral latent proteins that plays a key role in EBV-linked diseases. EBNA2 activates expression of both viral and host gene in part through interaction between its C-terminal acidic transactivation domain (TAD) and a number of host transcriptional regulatory proteins including the general transcription factor IIH (TFIIH) and the histone acetyltransferase CBP/p300. In this manuscript, we demonstrate that the TAD of EBNA2 binds to the pleckstrin homology (PH) domain from the Tfb1/p62 subunit of TFIIH and determine a three-dimensional structure of a complex between EBNA2 and Tfb1/p62. The structure shows that three hydrophobic residues from the TAD of EBNA2 make key interactions at the complex interface and these same residues also play an important role in the binding to CBP/p300. Comparison of the structure of the EBNA2-Tfb1 complex with complexes containing acidic TADs from other proteins (p53 and VP16) bound to the same Tfb1/p62 target highlights the inherent versatility of these intrinsically disordered domains and how minor variations in positioning of key hydrophobic residues allows them to bind to common targets using different functional interfaces.

## Introduction

The Epstein-Barr virus (EBV) is a double-stranded DNA-based virus that infects more than 90% of the world's adult population [1]. The high level of infectivity is due to the fact that the EBV can be easily transmitted through the exchange of saliva from an infected individual [2]. Once transmitted, the EBV infects epithelial cells and, in particular, B cells [3]. Upon primary infection of B cells, the EBV activates gene expression of proliferative elements in its latent phase [4]. During the latent phase, the host cells are slowly transformed to the point of immortalization, and this helps insure the persistence of the virus [5]. It is in this latent phase that most of the EBV-related diseases, including Hodgkin's lymphomas [6], [7], Burkitt's lymphomas [8], [9], nasopharyngeal carcinomas [10], [11] and post-transplant lymphoproliferative disorders [12], [13] are manifested.

Most of the EBV associated diseases are characterized by the presence of at least one of the nine viral latent proteins (review in [14]). These nine proteins include six nuclear antigens (EBNA), EBNA1, EBNA2, EBNA3A, EBNA3B, EBNA3C and EBNA-LP, and three latent membrane proteins (LMP), LMP1, LMP2A and LMP2B. Following primary infection with the EBV, all nine latent proteins are expressed in B-cells and this can lead to cellular immortalization [15]–[19]. The pathway to B-cell immortalization depends on a precise interplay between the EBV latent proteins and a number of different host factors [15]. In particular, it is known that EBNA2, EBNA3C and LMP1 are key elements for B-cells immortalization [16]–[18] and that EBNA2 plays an important role in regulating expression of the other two proteins [20]–[23].

EBNA2 is thus an essential latent protein required for B lymphocyte immortalization [16], [24] and its transforming capacity is directly linked to its ability to activate expression of both viral and host genes (5–9). Although EBNA2 does not contain a DNA-binding domain (DBD), it activates gene expression through a series of protein-protein interactions with host transcriptional regulatory proteins [25]. In order to activate transcription, EBNA2 must first bind to either the host CBF1/RBP-Jκ, or PU.1 proteins through interactions with its RBP-Jκ-binding domain (RBP-J) [26]–[29]. Once bound to either the CBF1/RBP-Jκ or PU.1 proteins, EBNA2 targets consensus sites in viral and host gene promoters using the DNA-binding domain of these host proteins [30]–[32]. In addition to forming protein-protein interactions with its RBP-J-binding domain, EBNA2 associates with a number of host transcriptional regulatory proteins, including general transcription factors (TFs), histone acetyl transferases (HATs) and chromatin remodelling complexes through interactions involving its C-terminal acidic transactivation domain (TAD; [33]–[35]).

It has been previously shown that the TAD of EBNA2 is between residues 431 and 487 at the extreme C-terminus of the protein [36]. This region of EBNA2 contains a number of acidic amino acids (Asp and Glu) as well as several hydrophobic/aromatic residues [37]. The amino acid composition of the TAD from EBNA2 is similar to a number of human and viral TADs, including the human tumor suppressor protein p53 (p53) and the herpes simplex viral protein 16 (VP16). Not surprisingly, the TAD of EBNA2 targets similar factors as other acidic TADs from viral and mammalian proteins. including the general transcription factor IIB (TFIIB; [35]), the p62/Tfb1 (human/yeast) subunit of the general transcription factor IIH (TFIIH; [33]), the TATA-binding protein-associated factor 40 (TAF40; [35]) and the histone acetyltransfereases (HAT) CBP (CREB-binding protein)/p300 [38]. These interactions involve the acidic TAD since mutating Trp458 to Thr (W458T mutant) within the TAD of EBNA2 disrupts its binding to TFIIB, TFIIH, TAF40 and CBP/p300 resulting in reduced transactivation activity [33], [35], [38].

Despite the essential role that interactions of the TAD of EBNA2 with host target proteins play in EBV infectivity, there are currently no detailed structural studies describing such interactions. In this manuscript, we structurally characterize the interaction of the acidic TAD of EBNA2 with the p62/Tfb1 subunit of TFIIH using nuclear magnetic resonance (NMR) spectroscopy. NMR chemical shift perturbation studies are used to define the minimal region from the intrinsically disordered TAD of EBNA2 required for binding to the pleckstrin homology (PH) domain located at the N-terminal of Tfb1 (Tfb1PH). NMR structure determination reveals formation of an α-helix within the TAD of EBNA2, and show that three hydrophobic residues within this helix make key interactions at the interface with Tfb1PH. Isothermal titration calorimetry (ITC) studies and transactivation studies in yeast using mutants of EBNA2 support the importance of these three hydrophobic residues for both the binding to Tfb1PH as well as the *in vivo* transactivation activity with the TAD of EBNA2. In addition, NMR chemical shift perturbation studies indicate that similar residues of EBNA2 are required for binding to the KIX domain of CBP/p300 (CBP KIX). This study provides the atomic level description of interactions involving the TAD of EBNA2 with target host proteins. In addition, comparison of the Tfb1PH-EBNA2_448–471_ complex with structures of the TAD of p53 and VP16 bound to Tfb1PH highlights the versatility of intrinsically disordered acidic TADs in recognizing common target host proteins.

## Results

### The TAD of EBNA2 binds to the PH domain of the Tfb1/p62 subunit of TFIIH

**Figure 1:**
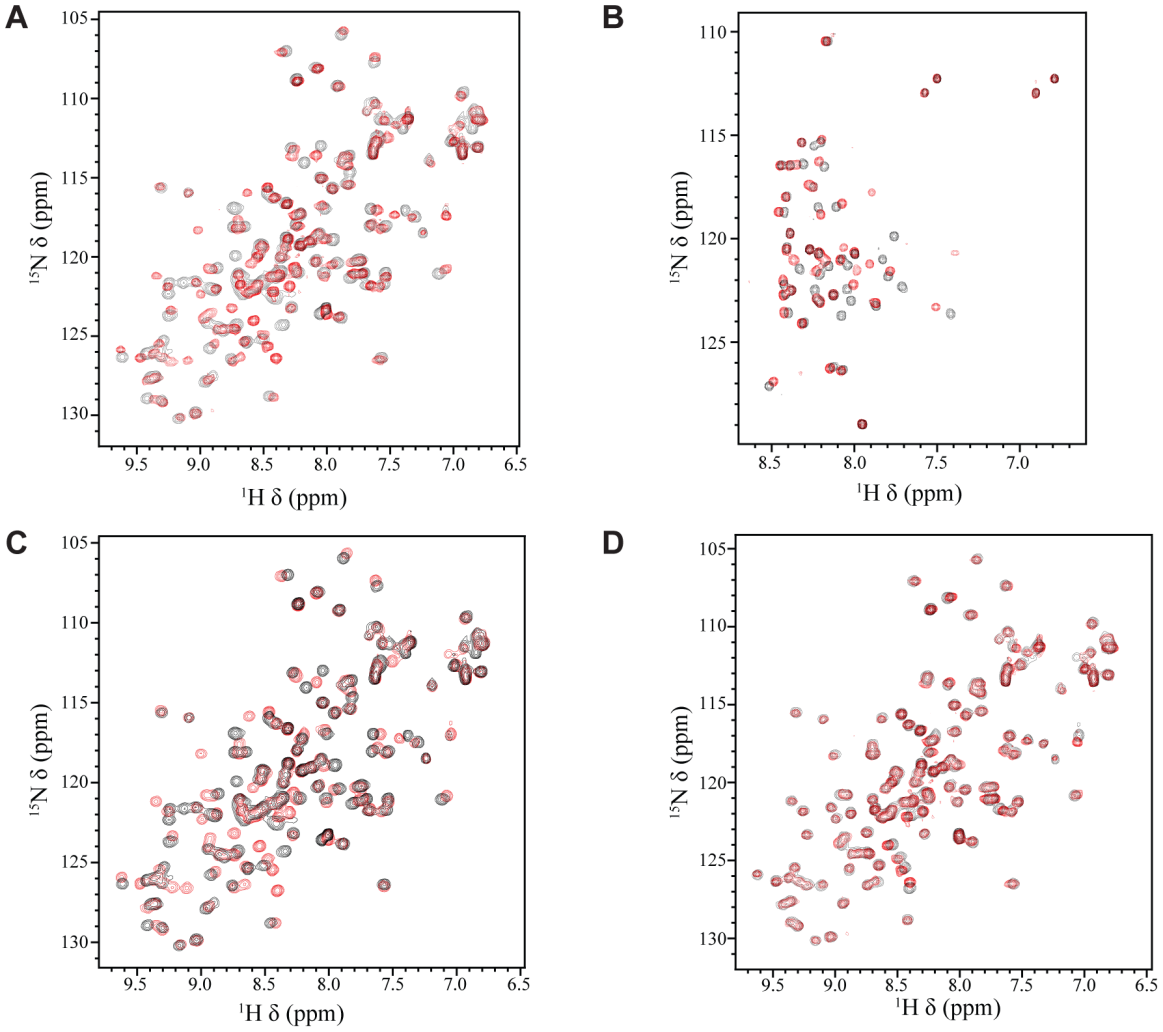
NMR titrations between Tfb1PH and the TAD of EBNA2. (**A**) Overlay of the ^1^H-^15^N HSQC spectra for a 0.5 mM sample of ^15^N-labeled Tfb1PH in the absence (black) or presence (red) of 1 mM unlabeled EBNA2_431–487_. (B) Overlay of the ^1^H-^15^N HSQC spectra for a 0.5 mM sample of ^15^N-labeled EBNA2_431–487_ in the absence (black) or presence (red) of 1 mM (red) unlabeled Tfb1PH. (**C**) Overlay of the ^1^H-^15^N HSQC spectra for a 0.5 mM sample of ^15^N-labeled Tfb1PH in the absence (black) or presence (red) of 1 mM unlabeled EBNA2_448–471_. (**D**) Overlay of the ^1^H-^15^N HSQC spectra for a 0.5 mM sample of ^15^N-labeled Tfb1PH in the presence of either 1 mM unlabeled EBNA2_448–471_ (black) or 1 mM unlabeled EBNA2_431–487_ (red).

It has been previously shown that EBNA2 interacts with the Tfb1/p62 subunit of TFIIH and that the interaction, *in vivo*, requires Trp458 within the TAD of EBNA2 [33]. In addition, we have previously shown that that the acidic TADs of p53 and VP16 bind to the PH domain of the Tfb1/p62 subunit of TFIIH [39]–[41]. To determine whether the PH domain of Tfb1 (Tfb1PH; residues 1–115 of Tfb1) interacts with the TAD of EBNA2 (EBNA2_431–487_; residues 431–487 of EBNA2) in a similar manner as the TADs of VP16 and p53, we performed NMR chemical shift perturbation studies. In the initial experiments, incremental additions of unlabeled EBNA2_431–487_ to ^15^N-labeled Tfb1PH cause significant changes in both the ^1^H and ^15^N chemical shifts for several signals of Tfb1PH in the ^1^H-^15^N HSQC spectrum (**Figure 1A** **and Supplementary Figure S1A**). When mapped onto the three-dimensional structure of Tfb1PH, the residues exhibiting significant chemical shift changes are located within the β5, β6 and β7 strands (**Supplementary Figure S1B**), and these changes are very similar to those observed when the TADs of p53 and VP16 bind to Tfb1PH [40], [41]. In the second set of experiments, incremental additions of unlabeled Tfb1PH to ^15^N-labeled EBNA2_431–487_ (**Figure 1B**) cause significant changes in both the ^1^H and ^15^N chemical shifts for several signals of EBNA2_431–487_ in the ^1^H-^15^N HSQC spectrum. In both sets of NMR experiments (**Figure 1A and 1B**), the chemical shift changes support the formation of an EBNA2_431–487_-Tfb1PH complex in intermediate to fast exchange on the NMR time scale. In addition, the experiments with the ^15^N-labeled EBNA2_431–487_ indicate that the TAD of EBNA2 is intrinsically disordered in the unbound state and that only 15–20 amino acids within this region are affected by the binding of Tfb1PH (**Figure 1B**).

### EBNA2_448–471_ is sufficient for binding to Tfb1PH

In an attempt to identify the minimal region of the EBNA2 TAD required for binding to Tfb1PH, a protein fragment comprised of residues 448–471 of EBNA2 (EBNA2_448–471_) was purified and analyzed for binding to Tfb1PH. This region of EBNA2 was selected based on the fact that Trp458 is required for binding to TFIIH *in vivo* [33]. To determine whether Tfb1PH interacts with EBNA2_448–471_ in a similar manner as EBNA2_431–487_, NMR chemical shift perturbation studies were performed by incremental addition of unlabeled EBNA2_448–471_ to ^15^N-labeled Tfb1PH. Virtually identical changes are observed in both the ^1^H and ^15^N chemical shifts of Tfb1PH in the ^1^H-^15^N HSQC spectrum when compared to the changes induced by EBNA2_431–487_ (**Figure 1 C–D** **and Supplementary Figure S1C–D**). These results demonstrate that EBNA2_448–471_ is sufficient for binding to Tfb1PH and support previous data demonstrating that the region including Trp458 is required for binding to TFIIH *in vivo* [33].

### EBNA2_448–471_ forms an α helix upon interaction with Tfb1PH

**Figure 2:**
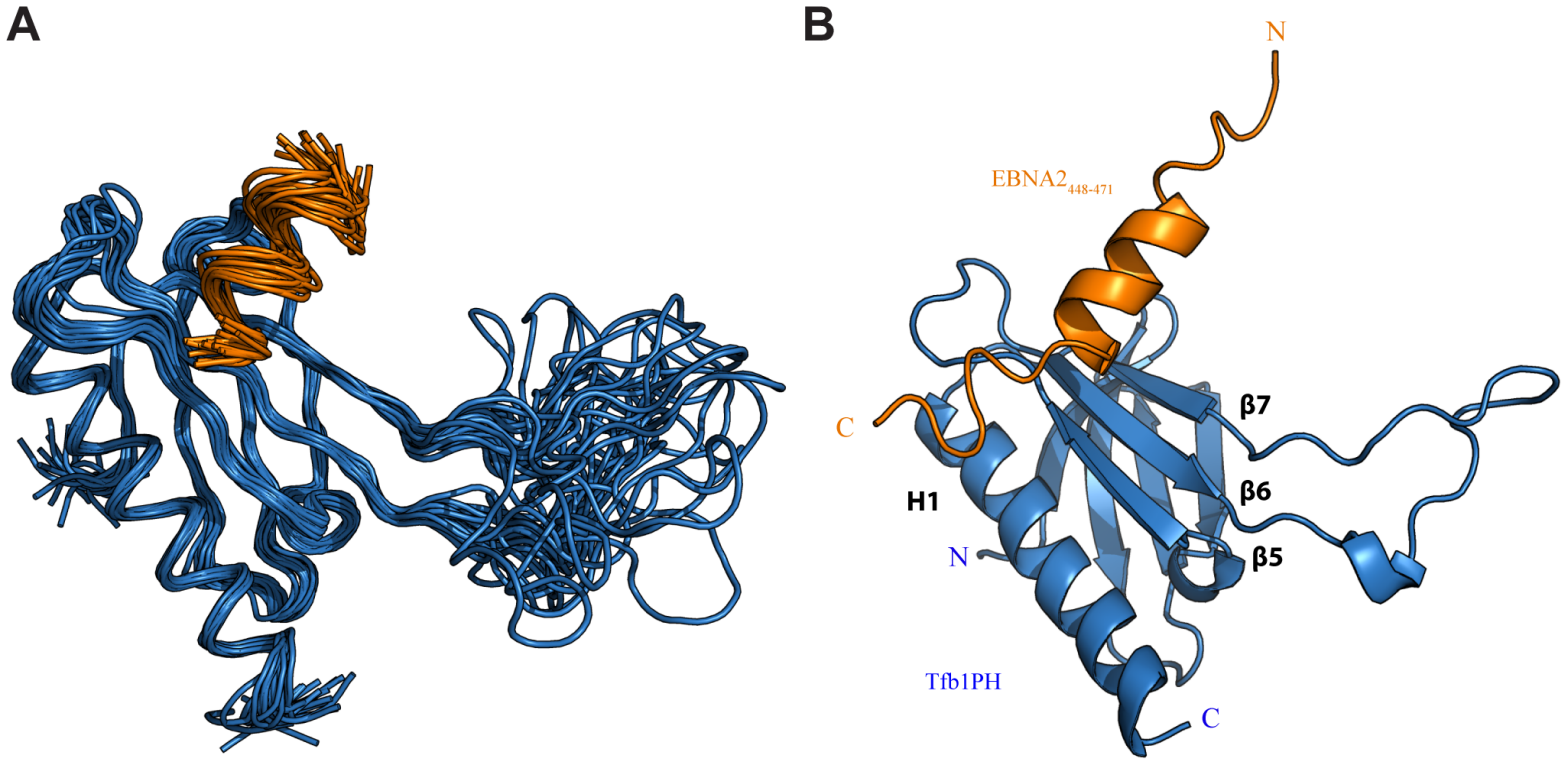
NMR structure of the Tfb1PH-EBNA2_448–471_ complex. (**A**) Overlay of the backbone trace of 20 structures of the complex between Tfb1PH (in blue) and EBNA2_448–471_ (in orange). The structures were superimposed using the backbone atoms C′, Cα and N of residues 4–63 and 86–112 of Tfb1PH and residues 454–464 of EBNA2_448–471_. (B) Ribbon model for the structure of the Tfb1PH-EBNA2_448–471_ complex.

Although the TAD of EBNA2 has been reported to interact with a number of transcriptional regulators [33]–[35], [38], there are currently no detailed structural studies characterizing the EBNA2 TAD in complex with one of its targets. Given that residues 448–471 of EBNA2 are sufficient for binding to Tfb1PH, we determined the three dimensional structure of a Tfb1PH-EBNA2_448–471_ complex by heteronuclear NMR. The structures of the Tfb1PH-EBNA2_448–471_ complex (**Figure 2A**) are well defined by the NMR data (**Table 1**). In addition, they are characterized by good backbone geometry, no significant restraint violations and low pairwise coordinate RMSD (root mean square deviation) values (**Table 1**). In complex with EBNA2_448–471_, the Tfb1PH structure (**Figure 2B**) is similar to its unbound form, which consists of a PH-domain fold containing a seven-stranded β sandwich (β1–β7) followed by a single α helix (H1; [39]). In complex with Tfb1PH, EBNA2_448–471_ forms a 9-residue α helix between Asp455 and Glu463 (**Figure 2B**), and this is consistent with results of the NMR chemical shift perturbation studies (**Figure 1B**).

**Table 1:** NMR and refinement statistics for Tfb1PH-EBNA2_448–471_ complex^a^.

	Tfb1PH-EBNA2_448–471_
**NMR distance and dihedral constraints**	
Number of distance constraints	
Total NOE	1753
Intra-residue	389
Inter-residue	
Sequential (|*i*−*j*| = 1)	379
Medium-range (|*i*−*j*|<4)	517
Long-range (|*i*−*j*|>5)	419
Intermolecular	49
Hydrogen bonds	20
Total dihedral angle restraints	145
φ	74
ψ	71
**Structure statistics**	
Violations (mean and s.d.)	
Distance constraints (Å)	0.003±0.002
Dihedral angle constraints (°)	0.03±0.03
Max. dihedral angle violation (°)	2.1
Max. distance constraint violation (Å)	0.21
Deviations from idealized geometry	
Bond lengths (Å)	0.0008±0.0001
Bond angles (°)	0.300±0.004
Impropers (°)	0.0.117±0.002
Atomic pairwise coordinate RMSD (Å)^b^	
Heavy atoms	1.20±0.12
Backbone atoms	0.67±0.11
Ramachandran statistics (%)^c^	
Residues in most favored regions	87.8
Residues in additional allowed regions	10.5
Residues in generously allowed regions	0.8
Residues in disallowed regions	0.9

a 20 conformers were selected for statistical analysis.

b Only residues 4–63 and 86–112 of Tfb1PH and residues 454–464 of EBNA2_448–471_ were used for the coordinate RMSD calculations.

c Based on PROCHECK-NMR analysis.

### The ΦXXΦΦ motif of EBNA2_448–471_ contributes key interactions with Tfb1PH

**Figure 3:**
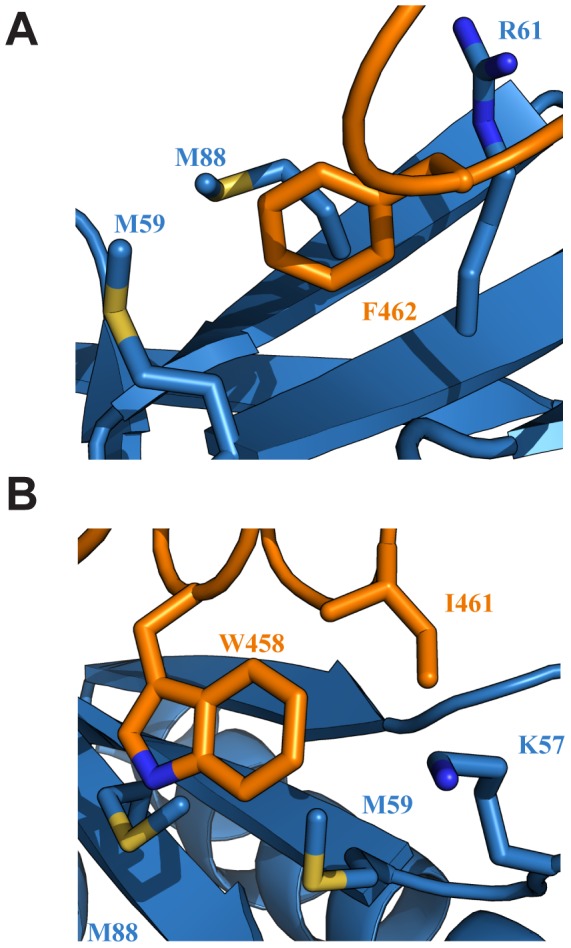
Key interactions at the interface of the Tfb1PH-EBNA2_448–471_ complex. (**A**) Ribbon representation of Tfb1PH (blue) and EBNA_448–471_ (orange) highlighting the side chains (shown in sticks) of Tfb1PH (M59, M88 and R61) that interact with the aromatic ring of Phe462 (F462) of EBNA_448–471_. (**B**) Ribbon representation of Tfb1PH (blue) and EBNA_448–471_ (orange) highlighting the side chains (shown in sticks) of Tfb1PH (M59, M88 and K57) that interact with the indole ring of Trp458 (W458) and the side chain of Ile461 (I461) of EBNA_448–471_.

In the Tfb1PH-EBNA2_448–471_ complex, EBNA2 binds to two shallow pockets on the surface of Tfb1PH, with three hydrophobic residues (Trp458, Ile461 and Phe462) on one face of the EBNA2 α-helix making important contributions to the binding interface (**Figure 3A–B**). The three hydrophobic residues are part of a ΦXXΦΦ motif (where Φ is hydrophobic/aromatic and X is any amino acid), a motif that is also found at the recognition interface of several acidic TADs, including p53 and VP16 (**Supplementary Figure S2**). The most significant contribution comes from Phe462 of EBNA2. In the Tfb1PH-EBNA2_448–471_ complex, the aromatic ring of Phe462 forms a cation-π interaction with the guanidinium group of Arg61 and van der Waals interactions with the side chains of Met59 and Met88 from Tfb1PH (**Figure 3A**). In addition to Phe462, Trp458 of EBNA2 also makes several important contributions to the binding interface with Tfb1PH. In the Tfb1PH-EBNA2_448–471_ complex, the indole ring of Trp458 makes van der Waals interactions with the side chains of K57, M59 and M88 as well as a potential cation-π interaction with the protonated amine group of K57 from Tfb1PH (**Figure 3B**). In addition to the interactions involving the two aromatic residues, Ile461 from the ΦXXΦΦ motif of EBNA2 forms van der Waals interactions with the side chains of Lys57 and Met59 from Tfb1PH (**Figure 3D**).

**Figure 4:**
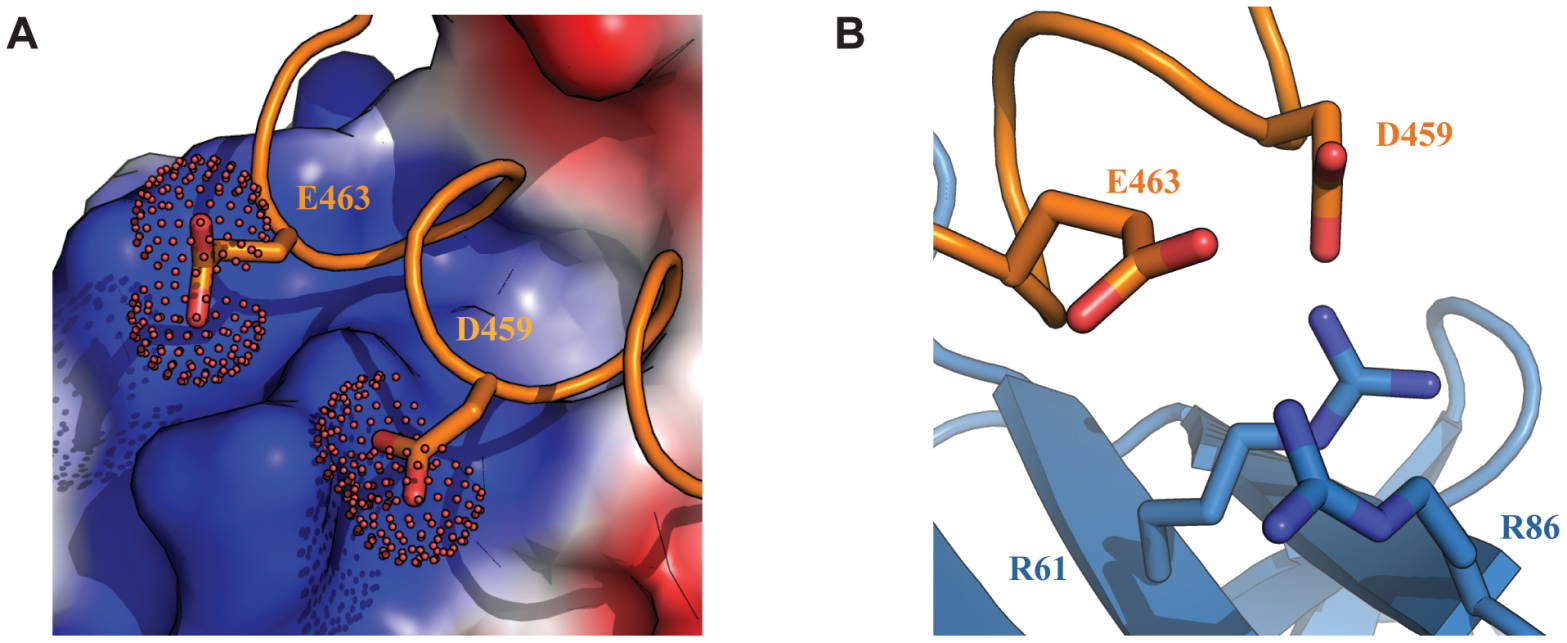
Electrostatic interactions at the interface of the Tfb1PH-EBNA2_448–471_ complex. (**A**) The interface of the Tfb1PH-EBNA2_448–471_ complex where Tfb1PH is shown as molecular surface with the electrostatic potential mapped on the surface (red negative potential and blue positive potential). EBNA_448–471_ is shown as a ribbon (orange) and the side chains of Asp459 (D459) and Glu463 (E463) are shown as sticks with the carboxyl group as a dotted surface. (**B**) Ribbon representation of Tfb1PH (blue) and backbone trace of the region of EBNA2_448–471_ (orange) highlighting the positively charged residues on the surface of Tfb1PH (R61 and R86) and the negatively charged residues of EBNA2 (D459 and E463) in positions to potentially form electrostatic interactions (shown as sticks).

Although a significant portion of the Tfb1PH-EBNA2_448–471_ interface is formed by interactions involving the three hydrophobic residues of the ΦXXΦΦ motif from EBNA2, several positively charged residues of Tfb1PH (Lys47, Lys57, Arg61, Arg86, Lys101 and Lys112) that surround the outer surfaces of the two binding pockets function to help position the negatively charged TAD of EBNA2 (**Figure 4A**). In particular, Arg61 and Arg86 of Tfb1PH are positioned to form electrostatic interactions with Asp459 and Glu463 of EBNA2 (**Figure 4B**).

### Lys57 and Arg61 of Tfb1PH are important for binding to the TAD of EBNA2

**Figure 5:**
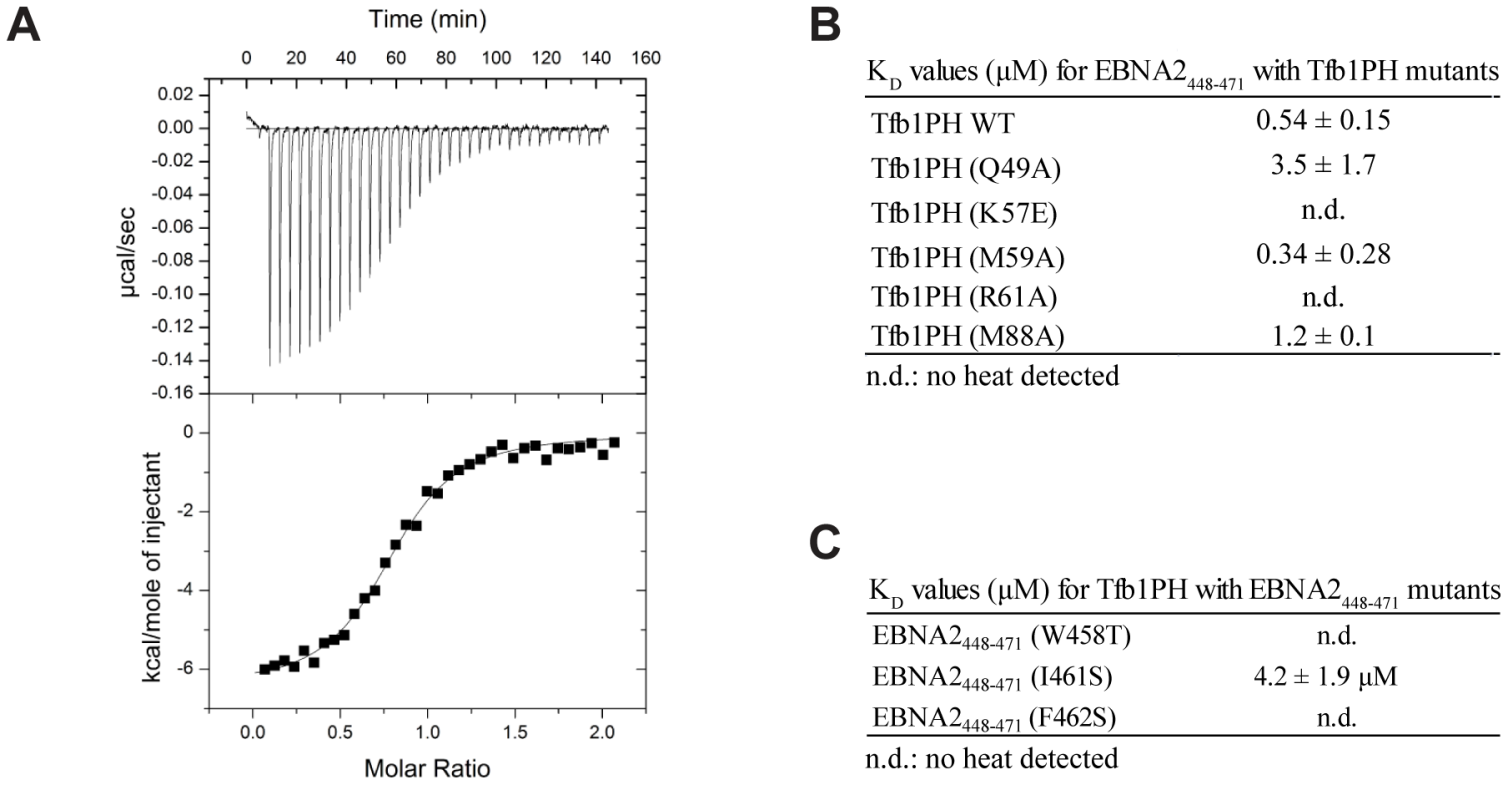
Dissociation constants of Tfb1PH-EBNA2_448–471_ and mutants. (**A**) Representative ITC thermogram obtained by successive addition of EBNA2_448–471_ to Tfb1PH. Experiments are performed at 25°C in a 20 mM Tris pH 7.4 and the results fit to a single-binding site model with 1∶1 stoichiometry. (**B**) Comparison of the dissociation constant (K_D_) values for the binding of Tfb1PH and its mutants (Q49A, K57E, M59A, R61A and M88A) to EBNA2_448–471_. (C) Comparison of the K_d_ values for the binding of EBNA2_448–471_ and its mutants (W458T, I461S and F462S) to Tfb1PH. In **B–C**, n.d. indicates that no heat of interaction was detected under the conditions tested for these mutants.

In order to assess the relative contributions of select residues of Tfb1PH in binding EBNA2, ITC studies were performed to determine the dissociation constants (K_D_) between EBNA2_448–471_ and five mutants of Tfb1PH [Tfb1PH (Q49A), Tfb1PH (K57E), Tfb1PH (M59A), Tfb1PH (R61E) and Tfb1PH (M88A) [**Figure 5**]. The altered residues in these five Tfb1PH mutants are all located on the surface of Tfb1PH that contacts EBNA2, and intermolecular NOEs are observed between these five residues of Tfb1PH and EBNA2. The five mutant proteins all fold in a similar manner as Tfb1PH and four of the five mutants (Q49A, K57E, R61E and M88A) displayed no heat of interaction with the TAD of p53 in ITC studies [40]. Only the M59A mutant displayed binding to the TAD of p53, but with significantly reduced affinity (K_D_ = 4.8±0.4 µM) compared to the wild-type Tfb1PH (K_D_ = 0.40±0.07 µM) [40]. In agreement with what has been observed for the TAD of p53, no heat of interaction is observed in ITC studies with either the R61E or the K57E mutants of Tfb1PH and the TAD of EBNA2 (**Figure 5B**) suggesting that the affinity is at least two orders of magnitude weaker than in comparison with the wild-type Tfb1PH (K_D_ = 0.54±0.15 µM). In contrast to what was observed for the TAD of p53, the M59A (K_D_ = 0.34±0.28 µM) mutant binds the TAD of EBNA2 with similar affinity, whereas the M88A (K_D_ = 1.2±0.1 µM) and the Q49A (K_D_ = 3.5±1.7 µM) mutants bind the TAD of EBNA2 with only a 2-fold and 6-fold respective drop in affinity relative to wild-type Tfb1PH. Taken together, these results show that although the TAD of EBNA2 binds to the same region of Tfb1PH as the TAD of p53, there are differences in how these two disordered TADs recognize a common target site in Tfb1.

### The ΦXXΦΦ motif of EBNA2 is crucial for binding Tfb1PH and transactivation

In the NMR structure of the Tfb1PH-EBNA2_448–471_ complex, Trp458, Ile461 and Phe462 within the ΦXXΦΦ motif of the TAD of EBNA2 make significant contributions to the interface with Tfb1PH. In the case of Trp458, the structure is consistent with previous studies showing that this residue is important for binding to TFIIH *in vivo* [33]. To assess the relative importance of these three residues of EBNA2 for binding to Tfb1PH, ITC experiments were performed to measure the relative K_D_'s of three EBNA2_448–471_ mutants (W458T, I461S and F462S) for Tfb1PH (**Figure 5**). As expected, all three EBNA2_448–471_ mutants displayed significantly weaker affinity for Tfb1PH (**Figure 5C**). No heat of interaction is detected with either the W458T or F462S mutant by ITC, whereas the binding to the I461S mutant is 8-fold weaker than to wild-type EBNA2_448–471_. These ITC results are consistent with both the *in vivo* data on the interaction of EBNA2 with TFIIH and the NMR structure of the Tfb1PH-EBNA2_448–471_ complex, and confirm that the two aromatic residues from the ΦXXΦΦ motif in the TAD of EBNA2 are particularly important for the interaction with Tfb1PH.

**Figure 6:**
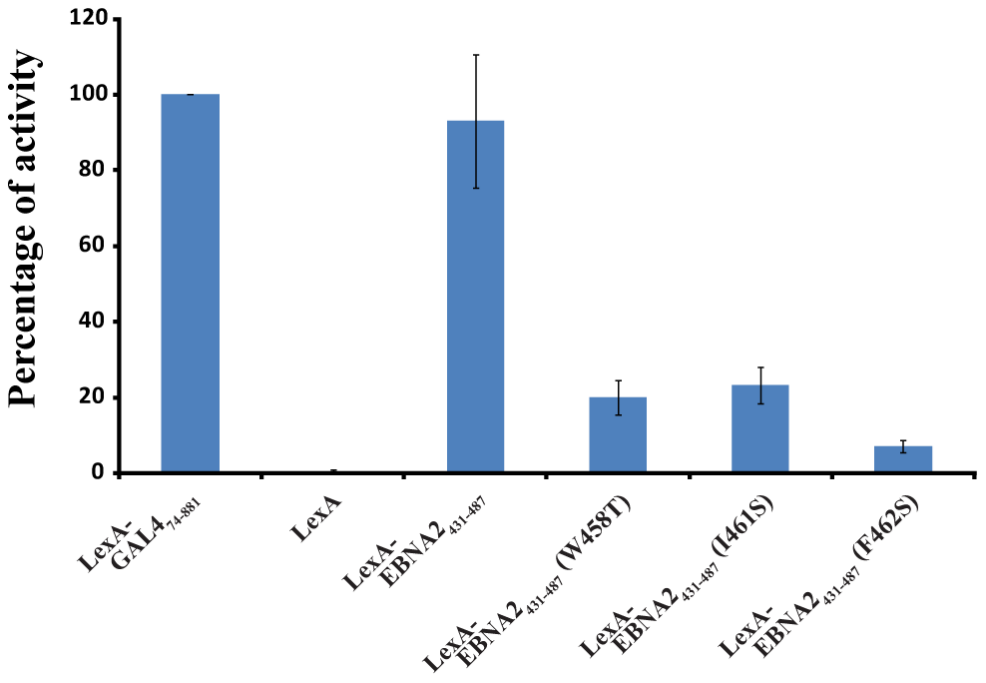
The hydrophobic residues of the ΦXXΦΦ motif from EBNA2 are important for transactivation. LexA-EBNA2_431–487_ and mutant (W458T, I461 and F462S) fusion proteins were co-transformed in yeast with the reporter LexA operator-Lac-Z fusion plasmid pSH18–34. Results are presented as the percentage of the β-galactosidase units of the tested fusion proteins relative to that of the LexA-GAL4_74–881_ positive control (100%). Error bars represent standard error about the mean of a minimum of three independent experiments.

EBNA2 plays an essential role in the transactivation of EBV latent genes, and it has been previously shown that Trp458 within the ΦXXΦΦ motif is important for this transactivation activity [33]. To assess the relative importance of Trp458, Ile461 and F462 in transactivation by EBNA2, an *in vivo* transactivation assay was performed in a yeast model system (**Figure 6**; [42]). For this assay, EBNA2_431–487_ and three related mutants (Trp458T, I462S and F462S) were fused to the DNA-binding domain (DBD) of LexA and their activity for a *lacZ* reporter gene was measured relative to a positive control (LexA-DBD-Gal4_74–881_; residues 74–881 of Gal4 fused to the LexA DBD). In this yeast assay, the LexA-DBD-EBNA2_431–487_ fusion protein activates transcription at 93±17% of the positive control, whereas the W458T mutant of EBNA2_431–487_ fused to the LexA-DBD activates at only 20±5%. In addition, both the I461S (23±5%) and F462S (7±2%) mutants of EBNA2_431–487_ fused to the LexA-DBD display reduced activity similar to the W458T mutant under these assay conditions. Consistent with the structure of the Tfb1PH-EBNA2_448–471_ complex and the ITC experiments, the three hydrophobic residues within the ΦXXΦΦ motif are important for the ability of the TAD of EBNA2 to activate transcription in a yeast model system.

### EBNA2 interacts with CBP/p300 in a similar fashion as with Tfb1PH

**Figure 7:**
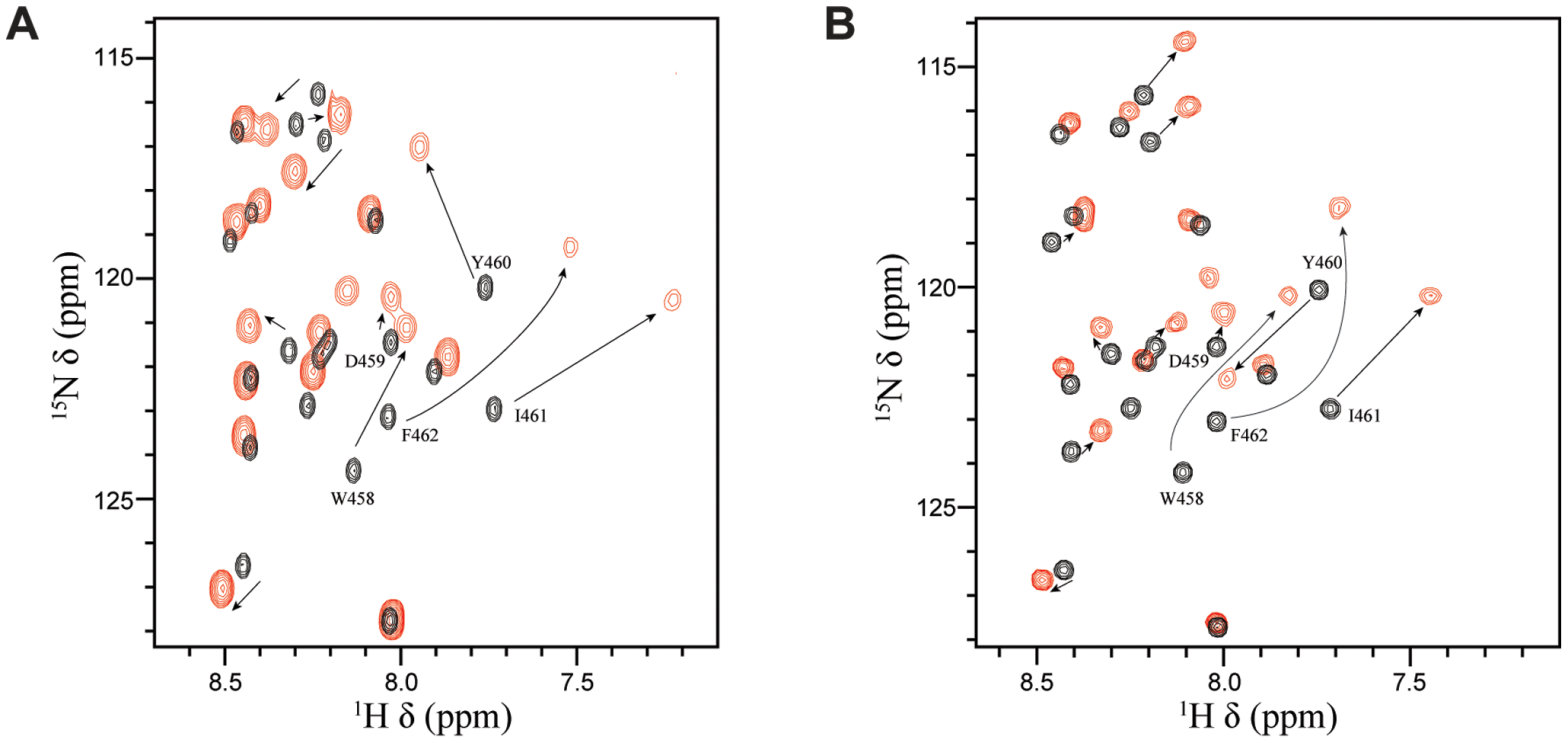
Tfb1PH and CBP KIX bind in a similar manner to EBNA2_448–471_. (**A**) Overlay of the ^1^H-^15^N HSQC spectra for a 0.5 mM sample of ^15^N-labeled EBNA2_448–471_ in the absence (black) or presence (red) of 0.5 mM unlabeled Tfb1PH. (**B**) Overlay of the ^1^H-^15^N HSQC spectra for a 0.5 mM sample of ^15^N-labeled EBNA2_448–471_ in the absence (black) or presence (red) of 0.5 mM unlabeled CBP KIX.

Numerous transcriptional regulatory proteins containing acidic TADs have been shown to interact with the homologous histone acetyl transferases (HATs), CBP (CREB-binding protein) and p300 (CBP/p300), and these interactions are required for their ability to activate gene expression [38]. In the case of p53, the regulation is quite complex. Four different domains of CBP/p300 have been shown to interact with the TAD of p53 in a phosphorylation-dependent manner, including the TAZ1/CH1 domain, the KIX domain (CBP KIX), the TAZ2/CH3 domain and the IBID domain [43], [44]. Interestingly, it has been previously demonstrated that EBNA2 can interact with both the N-terminus and the C-terminus of CBP/p300 and that the W458T mutation within the acidic TAD disrupts these interactions [38]. To determine whether the same residues of EBNA2 that interact with Tfb1PH are also involved in binding to CBP/p300, NMR chemical shift perturbation studies were performed with EBNA2_448–471_ and CBP KIX (residues 586–672 of human CBP). Addition of unlabeled CBP KIX to a ^15^N-labeled EBNA2_448–471_ causes significant changes in both the ^1^H and ^15^N chemical shifts of several signals from EBNA2. The signals displaying the most significant changes in the ^1^H-^15^N-HSQC spectra belongs to residues composing the ΦXXΦΦ motif of EBNA2 and are very similar to those seen with Tfb1PH (**Figure 7** **and Supplementary Figure S3**). The results clearly show that the ΦXXΦΦ motif of EBNA2 is important for the interaction with CBP KIX and suggest that the binding interface resembles the one formed between EBNA2 and Tfb1PH.

## Discussion

EBNA2 activates expression of both viral and host genes in part through the ability of its acidic TAD to participate in a series of protein-protein interactions with several host transcriptional regulatory proteins, including the Tfb1/p62 subunit of TFIIH and CBP/p300 [15], [33], [35], [38]. In this manuscript, we have structurally characterized the interactions of the TAD of EBNA2 with these two host target proteins. NMR chemical shift perturbation studies demonstrate that the TAD of EBNA2 binds to the PH domain of Tfb1 (Tfb1PH), and that residues 448–471 of EBNA2 (EBNA2_448–471_) are both required and sufficient for the interaction. Structural determination of a Tfb1PH-EBNA2_448–471_ complex indicates that EBNA2_448–471_ transitions from an intrinsically disordered state to a 9-residue α helix between Asp455 and Glu463 upon binding to Tfb1PH. Within this 9-residue helix of EBNA2_448–471_, three hydrophobic amino acids (Trp458, Ile461 and Phe462) that form a ΦXXΦΦ motif make a series of key interactions at the interface with Tfb1PH. In addition, there are two potential electrostatic interactions at the interface of the complex involving negatively charged residues from EBNA2 and positively charged residues of Tfb1PH. Mutational studies of the three key hydrophobic residues from the ΦXXΦΦ motif indicate that they are all important for transactivation in a yeast model system. In addition, NMR chemical shift perturbation studies strongly suggest that the same residues of EBNA2 involved in binding Tfb1PH are also required for binding to the KIX domain of CBP.

Previous *in vivo* studies have shown that EBNA2 is essential for immortalization of B-cell following EBV infection and that viruses expressing a W458T mutant of EBNA2 lose the ability to transform host cells [45]. Unlike the wild-type protein, the W458T EBNA2 mutant fails to interact with a number of transcriptional regulatory factors including TFIIB, TAF40 TFIIH, CBP and PCAF [33]–[35], [38] and this likely explains the decreased infectivity associated with the mutant viruses. In agreement with these studies, our experiments demonstrate that Trp458 is the first hydrophobic residue of a ΦXXΦΦ motif that is important for binding to both Tfb1PH and CBP KIX. The NMR structure of the Tfb1PH-EBNA2_448–471_ complex shows that Trp458 plays a key role at the interface with Tfb1PH, and this is supported by a loss of binding in ITC studies and diminished transactivation activity in yeast with the W458T mutant. In addition, the structural characterization of the Tfb1PH-EBNA2_448–471_ complex reveals that Phe462 maybe more important than Trp458, as it appears to dictate the location of the ΦXXΦΦ motif on the surface to Tfb1PH through formation of a key cation-π interaction with Arg61 of Tfb1PH. The importance of Phe462 is supported by ITC and yeast activation studies with the F462S mutant of EBNA2 as well as ITC studies with the R61A mutant of Tfb1PH. Therefore, it is likely that Phe462 is also a key residue in EBNA2 function.

Like EBNA2, several other transcription factors containing acidic TADs have been shown to bind to the Tfb1/p62 subunit of TFIIH, and this interaction is crucial to their ability to activate expression of target genes [33], [46]–[49]. Previously, we have shown that both p53 and VP16 also bind to TFIIH through interactions between the PH domain of Tfb1/p62 and a small region within their acidic TAD [40], [41]. Like the TAD of EBNA2, the intrinsically disordered TADs of p53 and VP16 both form 9-residue α helices upon binding Tfb1PH that contains an important ΦXXΦΦ motif (**Supplementary Figure S2**). For all three TADs, a Phe residue is located at the third hydrophobic position of the motif, and the residue likely functions as the anchor point for the helix on Tfb1PH. This is consistent with previous studies showing that substituting for Phe residues within several acidic TADs dramatically reduces their ability to activate transcription [40], [50], [51].

**Figure 8:**
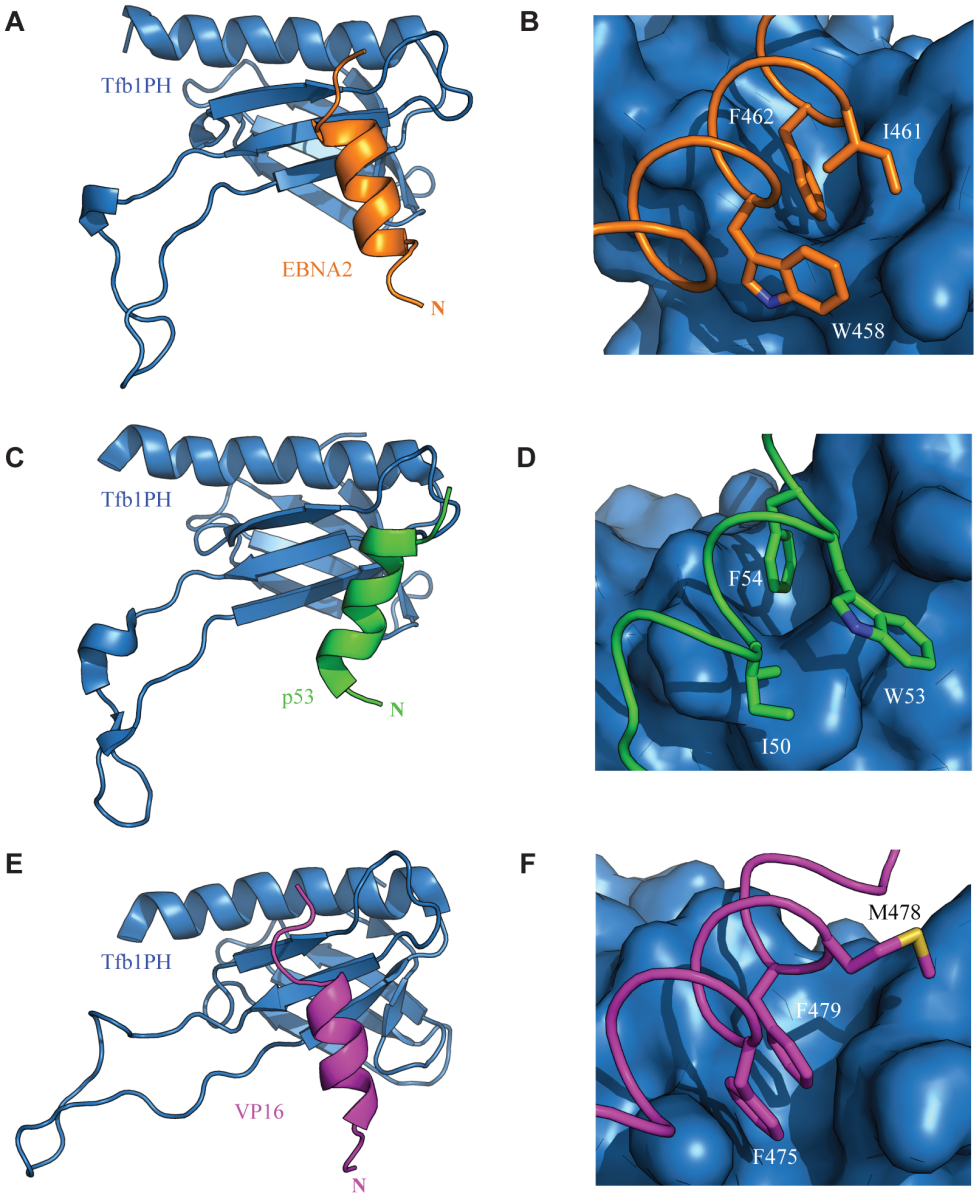
Comparison of the TADs of p53, VP16 and EBNA2 in complex with Tfb1PH. The structure of Tfb1PH (blue) is shown as either a ribbon (**A, C and E**) or molecular surface (**B, D and F**) in complex with the TADs of EBNA2, p53 and VP16, In **A**–**B**, the TAD of EBNA2 is shown as a ribbon (orange). The complete helix of EBNA2 is shown in **A**, whereas the three key hydrophobic residues of the ΦXXΦΦ motif (W458, I461 and F462) of EBNA2 are highlighted in stick (orange) on the surface of Tfb1PH in **B**. In **C**–**D**, the TAD p53 is shown as a ribbon (green). The complete helix of p53 is shown in **A**, whereas and the three key hydrophobic residues of the ΦXXΦΦ motif (I50, W53 and F54) of p53 are highlighted in stick (green) on the surface of Tfb1PH in **D**. In **E–F**, the TAD VP16 is shown as a ribbon (magenta). The complete helix of VP16 is shown in **E**, whereas the three key hydrophobic residues of the ΦXXΦΦ motif (F475, M478 and F479) of VP16 are highlighted in stick (magenta) on the surface of Tfb1PH in **F**.

Although the acidic TADs of p53, VP16 and EBNA2 all bind to the same region of Tfb1PH, the composition and spatial arrangement of the first and second hydrophobic residue within the ΦXXΦΦ motifs differs in all three cases. These two hydrophobic residues appear to help establish the location of key negatively charged residues in the TAD so that they are in position to form electrostatic interactions with positively charged amino acids in Tfb1PH (**Figure 8** **and Supplementary Figure S4**). Each complex contains two key electrostatic interactions involving negatively charged residues from the TADs, but the interactions with the TAD of EBNA2 are slightly different than those seen with p53 and VP16. In all three cases, a negatively charged residue located at the second position of the ΦXXΦΦ motif forms an electrostatic interaction with Arg61 of Tfb1PH. For both p53 and VP16, this residue is a Glu whereas for EBNA2 this residue is an Asp. For the second electrostatic interaction, the first residue after the ΦXXΦΦ motif (ΦXXΦΦE) in EBNA2 (Glu463) interacts with Arg86 from Tfb1PH. In contrast, the second electrostatic interaction observed with p53 and VP16 occurs between a Glu residue located at the second position after the ΦXXΦΦ motif (ΦXXΦΦXE) and Lys57 from Tfb1PH.

One of the key features observed for p53 is that phosphorylation of Ser46 in p53 enhances its affinity for Tfb1PH/p62PH by forming an additional electrostatic interaction with a lysine side chain located in the loop linking β1 and β2 strands [40]. Ser46 is the residue immediately prior to the first residue of the helix in p53. There is no equivalent phosphorylation site in VP16, but it contains a negatively charged residue in the first position of the helix that helps to stabilize the α helix through a favourable interaction with the helix dipole [41], [52], [53]. Like VP16, there are no apparent phosphorylation sites located at the N-terminus of the EBNA2 α-helix, and there is a negatively charged residue in the first position of the helix. Thus, it appears that binding of the two viral activators to Tfb1PH/p62PH is not controlled by phosphorylation as is seen with the host p53 protein.

In conclusion, our results provide a structural view of how EBNA2 usurps the host transcription factors TFIIH and CBP/p300 to modulate viral and host gene expression. In addition, they provide insights into the role of TFIIH and CBP in EBNA2 function as well as supporting the key role of Trp458 in EBNA2 activation. The structure of the Tfb1PH-EBNA2_448–471_ complex together with the ITC and transactivation studies with mutant proteins, point to a key role for Phe462 in EBNA2 function and indicates that it plays a similar role to the analogous residue in other acidic TADs such as p53 and VP16. In addition, this study highlights the inherent versatility of disordered acidic TADs and, particularly how minor variations in the position of hydrophobic and acidic residues allows them to form distinct structural interfaces with a common target protein.

## Materials and Methods

### Plasmid construction

The cDNA encoding Tfb1PH (residues 1–115 of Tfb1) was cloned into the pGEX-2T vector (GE Healthcare) as previously described [39]. The plasmid for expressing the CBP KIX (residues 586–672 of CBP) as a His-tag fusion protein was kindly provided by Dr. Alana Schepartz (Yale University). The cDNA for EBNA2_431–487_ was kindly provided by Dr. S. Diane Hayward (John Hopkins University) and cloned into the pGEX-2T vector. The cDNA for the expression of EBNA2_448–471_ was synthetically prepared (BioCorp) and cloned into the pGEX-2T vector. The Tfb1PH and EBNA2_448–471_ mutants were generated using the QuikChange II site-directed mutagenesis procedure (Stratagene) starting from the wild-type sequence cloned in the pGex-2T vector. The plasmid for expressing the LexA-EBNA2_431–487_ fusion protein was prepared by inserting the EcoRI-BamHI-digested PCR product generated from the corresponding pGEX-2T expression vector into the EcoRI and BamHI sites of the AB-426 vector [54]. The LexA-EBNA2_431–487_ mutants were generated using the QuikChange II site-directed mutagenesis procedure starting from the wild-type sequence cloned in the AB-426 vector.

### Protein expression and purification

Tfb1PH and CBP KIX were expressed and purified as previously described [39], [55]. EBNA2_431–487_, EBNA2_448–471_ and mutants were expressed as GST fusion proteins in *E. coli* host strain TOPP2, purified over glutathione-sepharose resin (GE Healthcare) and cleaved with thrombin (Calbiochem) as previously described [39]. Following cleavage, EBNA2 peptides were purified over a High Performance Q-Sepharose (GE Healthcare) column and dialyzed into appropriate buffers for isothermal titration calorimetry (ITC) and nuclear magnetic resonance (NMR) studies. Uniformly (>98%) ^15^N-labeled and ^15^N/^13^C-labeled proteins were prepared in M9-minimal media containing ^15^NH_4_Cl (Sigma) and/or ^13^C_6_-glucose (Sigma) as the sole nitrogen and carbon sources.

### ITC experiments

ITC titrations were performed as described [56], at 25°C in 20 mM Tris-HCl buffer (pH 7.5). All titrations fit a single-binding site equation with 1∶1 stoichiometry and K_D_ values are the average of two or more separate experiments.

### NMR experiments

For chemical shift perturbation studies with Tfb1PH, either unlabeled EBNA2_448–471_ or unlabeled EBNA2_431–487_ was added in stepwise increments to a sample containing 0.5 mM ^15^N-Tfb1PH in 20 mM sodium phosphate buffer (pH 6.5) in 90% H_2_O/10% D_2_O. The same experimental conditions were used for the reverse experiments with the exception that unlabeled Tfb1PH was incrementally added to either ^15^N-EBNA2_448–471_ or ^15^N-EBNA2_431–487_. For chemical shift perturbation studies with CBP KIX, unlabeled CBP KIX was incrementally added to either 0.5 mM ^15^N-EBNA2_448–471_ or 0.5 mM ^15^N-EBNA2_431–487_ in 20 mM sodium phosphate buffer (pH 6.5) in 90% H_2_O/10% D_2_O. For the NMR structural studies of the Tfb1PH-EBNA2_448–471_ complex, four different samples were used. The first two samples contained 0.5 mM of either ^15^N- or ^15^N/^13^C-Tfb1PH and 1.5 mM unlabeled EBNA2_448–471_, whereas the other two samples contained 0.5 mM of either ^15^N- or ^15^N/^13^C-EBNA2_448–471_ and 1.5 mM of unlabeled Tfb1PH. All samples were in 20 mM sodium phosphate (pH 6.5) in either 90% H_2_O/10% D_2_O or 100% D_2_O, 1 mM EDTA and 1 mM DTT. NMR experiments were carried out at 300 K on Varian Unity Inova 500, 600 and 800 MHz spectrometers equipped with z pulsed-field gradient units and triple resonance probes. For the chemical shift perturbation studies, 2D ^1^H-^15^N HSQC experiments were performed. For structure determination of the Tfb1PH-EBNA2_448–471_ complex, the backbone and aliphatic side chain resonances (^1^H, ^15^N, ^13^C) were assigned as previously reported [57]. Interproton distance restraints were measured from 3D ^15^N-edited NOESY-HSQC and ^13^C-edited HSQC-NOESY spectra with a τ_m_ = 90 ms. Intermolecular distance restraints were obtained from 3D ^15^N/^13^C F_1_-filtered, F_3_-edited NOESY experiment with a τ_m_ = 90 ms [58], [59]. NMR data were processed with NMRPipe/NMRDraw [60] and analyzed with Analysis from the CCPNMR suite [61].

### Structure calculations

The NOE-derived distance restraints were divided into four classes defined as strong (1.8–2.8 Å), medium (1.8–4.0 Å), weak (1.8–5.0 Å) and very weak (3.3–6.0 Å). NOE constraints involving equivalent or non stereo chemically-assigned protons were corrected using the Fletcher method [62]. Backbone dihedral angle restraints for EBNA2_448–471_ were generated with DANGLE of the CCPNMR suite [61] and for Tfb1PH with TALOS-N [63]. Hydrogen-bond restraints were identified from the non-exchanged H_N_ signals in a ^1^H-^15^N HSQC recorded in 100% D2O. The structure of the Tfb1PH-EBNA2_448–471_ complex was calculated using the program CNS [64] using a combination of torsion angle dynamics and Cartesian dynamics. Starting from an extended structure with standard geometry, 100 conformers were calculated satisfying all the experimental restraints with no NOE violation greater than 0.2 Å and no dihedral angle violations greater than 2°. The quality of the structures was verified with PROCHECK-NMR [65] and MOLMOL [66]. All figures were generated with PyMol [67].

### ß-galactosidase activation assay

ß-galactosidase assays were performed as previously described [42]. Results are presented as the mean of the percentages obtained by the ß-galactosidase units of the tested LexA-fusion proteins on the mean of the ß-galactosidase units of the LexA-GAL4 positive control ± standard error of the mean (SEM). Western blot analysis was performed with an anti-LexA antibody to verify expression of all the LexA-fusion proteins.

### Accession codes

Protein Data Bank: 2MKR. BMRB: 19791

## Supporting Information

Figure S1 **NMR chemical shift perturbation studies of Tfb1PH with either EBNA2_448–471_ or EBNA2_431–487_.** (**A** and **C**) Histogram showing the variation in chemical shifts observed in the ^1^H-^15^N HSQC spectra of ^15^N-labeled Tfb1PH following the addition of either (**A**) EBNA2_431–487_ or (**C**) EBNA2_448–471_. Changes in chemical shift values are represented by Δδ = [(0.17ΔN_H_)^2^+(ΔH_N_)^2^]^1/2^, where ΔN_H_ and ΔH_N_ is the difference in chemical shift between the two signals in ppm. (**B** and **D**) Ribbon model of the structure of Tfb1PH (blue; PDB code 1Y5O) with orange highlights for the amino acids of ^15^N-labeled Tfb1PH showing a significant chemical shift change (Δδ>0.1 ppm) upon formation of (**B**) the Tfb1PH-EBNA2_431–487_ complex or (**D**) theTfb1PH-EBNA2_448–471_ complex.
(PDF)

Figure S2 **Tfb1/p62 binding regions of acidic TADs.** Sequence alignment of the regions from the TADs of p53, VP16 and EBNA2 (that form α helices when bound to the Tfb1/p62 subunit of TFIIH. Key hydrophobic residues (Φ) of the ΦXXΦΦ motif that directly interact with Tfb1PH/p62PH are highlighted in black. Numbers of residues shown on each side of the sequence are inclusive.
(PDF)

Figure S3 **NMR mapping studies of EBNA2_448–471_ with Tfb1PH and CBP KIX.** Histogram showing the variation in chemical shifts observed in the ^1^H-^15^N HSQC spectra of ^15^N-labeled EBNA2_448–471_ following the addition of either (**A**) Tfb1PH or (**B**) CBP KIX. Changes in chemical shifts are represented by Δδ = [(0.17ΔN_H_)^2^+(ΔH_N_)^2^]^1/2^, where ΔN_H_ and ΔH_N_ is the chemical shift difference between the two signals in ppm.
(PDF)

Figure S4 **Comparison of the TADs of p53, VP16 and EBNA2 in complex with Tfb1PH.** The interface of the Tfb1PH in complex with the TAD of EBNA2, p53 and VP16, where Tfb1PH is shown as molecular surface (blue), In **A**–**B**, the TAD of EBNA2 is shown as a ribbon (orange) and the three key hydrophobic residues of the ΦXXΦΦ motif (W458, I461 and F462) of EBNA2 are shown in stick (orange). In **A**, the view is from the C-terminus of the EBNA2 helix. In **B**, the view is from the N-terminus of the EBNA2 helix. In **C**–**D**, the TAD p53 is shown as a ribbon (green) and the three key hydrophobic residues of the ΦXXΦΦ motif (I50, W53 and F54) of p53 are shown in stick (green). In **C**, the view is from the C-terminus of the p53 helix. In **D**, the view is from the N-terminus of the p53 helix. In **E–F**, the TAD VP16 is shown as a ribbon (magenta) and the three key hydrophobic residues of the ΦXXΦΦ motif (F475, M478 and F479) of VP16 are shown in stick (magenta). In **E**, the view is from the C-terminus of the VP16 helix. In **F**, the view is from the N-terminus of the VP16 helix.
(PDF)
