# Supplementary material for: Structural and Functional Characterization of a Complex between the Acidic Transactivation Domain of EBNA2 and the Tfb1/p62 Subunit of TFIIH
Source: PLoS Pathog. 2014 Mar 27;10(3):e1004042. doi: 10.1371/journal.ppat.1004042 (PMC3968163; doi:10.1371/journal.ppat.1004042)
Supplement: Figure S1 — NMR chemical shift perturbation studies of Tfb1PH with either EBNA2448–471 or EBNA2431–487. (A and C) Histogram showing the variation in chemical shifts observed in the 1H-15N HSQC spectra of 15N-labeled Tfb1PH following the addition of either (A) EBNA2431–487 or (C) EBNA2448–471. Changes in chemical shift values are represented by Δδ = [(0.17ΔNH)2+(ΔHN)2]1/2, where ΔNH and ΔHN is the difference in chemical shift between the two signals in ppm. (B and D) Ribbon model of the structure of Tfb1PH (blue; PDB code 1Y5O) with orange highlights for the amino acids of 15N-labeled Tfb1PH showing a significant chemical shift change (Δδ>0.1 ppm) upon formation of (B) the Tfb1PH-EBNA2431–487 complex or (D) theTfb1PH-EBNA2448–471 complex. (PDF) [file ppat.1004042.s001.pdf]

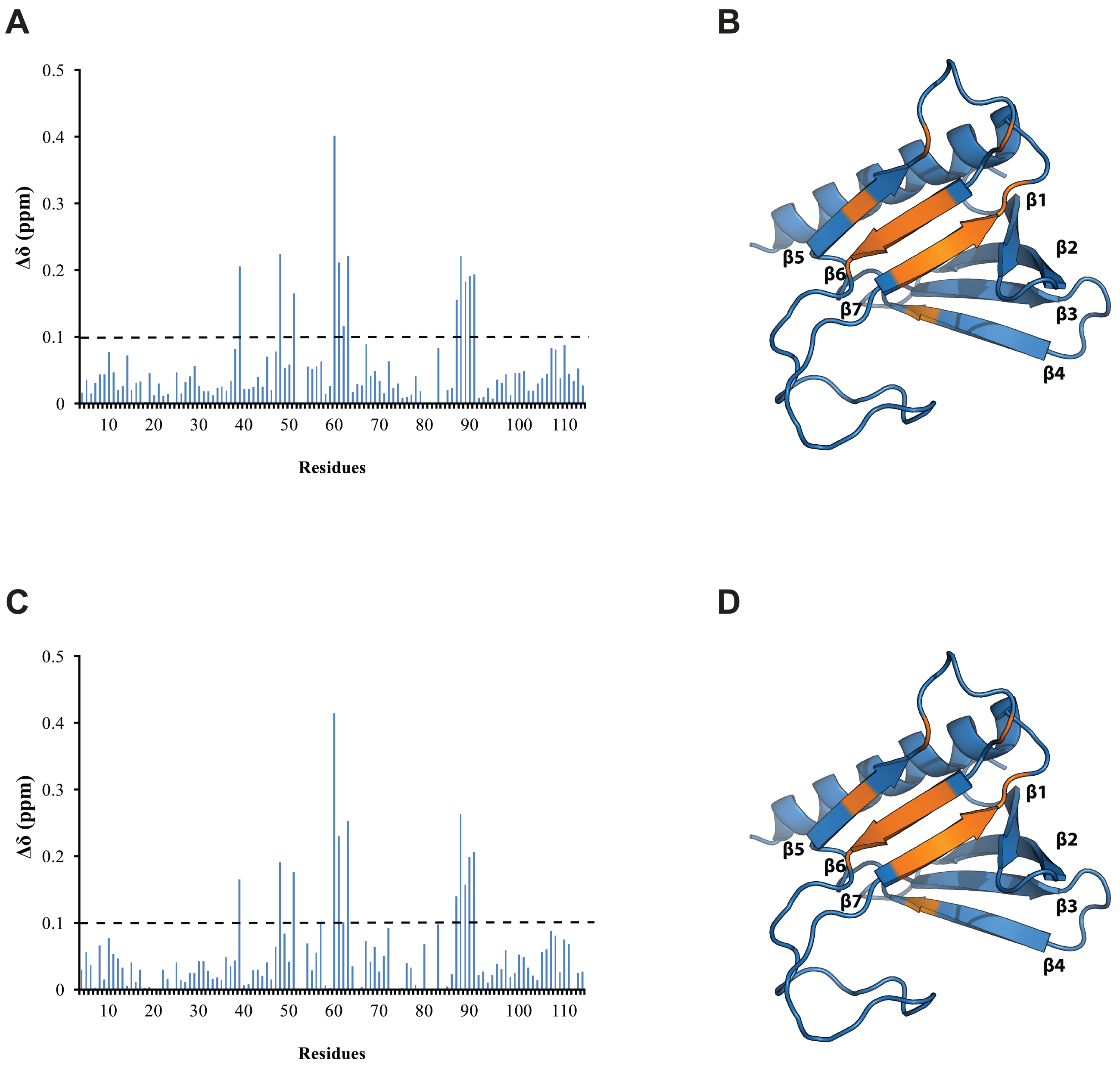

Supplementary Figure S1. NMR chemical shift perturbation studies of Tfb1PH with either EBNA2$_{448\text{-}471}$ or EBNA2$_{431\text{-}487}$. (**A** and **C**) Histogram showing the variation in chemical shifts observed in the ^{1}H-^{15}N HSQC spectra of ^{15}N-labeled Tfb1PH following the addition of either (**A**) EBNA2$_{431\text{-}487}$ or (**C**) EBNA2$_{448\text{-}471}$. Changes in chemical shift values are represented by $\Delta\delta = [(0.17\Delta N_H)^2 + (\Delta H_N)^2]^{1/2}$, where ΔN_H and ΔH_N is the difference in chemical shift between the two signals in ppm. (**B** and **D**) Ribbon model of the structure of Tfb1PH (blue; PDB code 1Y5O) with orange highlights for the amino acids of ^{15}N-labeled Tfb1PH showing a significant chemical shift change ($\Delta\delta > 0.1$ ppm) upon formation of (**B**) the Tfb1PH-EBNA2$_{431\text{-}487}$ complex or (**D**) theTfb1PH-EBNA2$_{448\text{-}471}$ complex.
