# Supplementary material for: Structural and Functional Characterization of a Complex between the Acidic Transactivation Domain of EBNA2 and the Tfb1/p62 Subunit of TFIIH
Source: PLoS Pathog. 2014 Mar 27;10(3):e1004042. doi: 10.1371/journal.ppat.1004042 (PMC3968163; doi:10.1371/journal.ppat.1004042)
Supplement: Figure S2 — Tfb1/p62 binding regions of acidic TADs. Sequence alignment of the regions from the TADs of p53, VP16 and EBNA2 (that form α helices when bound to the Tfb1/p62 subunit of TFIIH. Key hydrophobic residues (Φ) of the ΦXXΦΦ motif that directly interact with Tfb1PH/p62PH are highlighted in black. Numbers of residues shown on each side of the sequence are inclusive. (PDF) [file ppat.1004042.s002.pdf]

EBNA2	(452)	ADLDES	W	DY	IF	ETTE	(466)
VP16	(469)	DMADFE	F	EQ	MF	TDAL	(483)
P53	(44)	MLSPDD	I	EQ	WF	TEDP	(58)
			Φ	XX	ΦΦ		

Supplementary Figure S2. Tfb1/p62 binding regions of acidic TADs.
Sequence alignment of the regions from the TADs of p53, VP16 and EBNA2 (that form α helices when bound to the Tfb1/p62 subunit of TFIIH. Key hydrophobic residues (Φ) of the $\Phi XX\Phi\Phi$ motif that directly interact with Tfb1PH/p62PH are highlighted in black. Numbers of residues shown on each side of the sequence are inclusive.
