# Supplementary material for: Structural and Functional Characterization of a Complex between the Acidic Transactivation Domain of EBNA2 and the Tfb1/p62 Subunit of TFIIH
Source: PLoS Pathog. 2014 Mar 27;10(3):e1004042. doi: 10.1371/journal.ppat.1004042 (PMC3968163; doi:10.1371/journal.ppat.1004042)
Supplement: Figure S3 — NMR mapping studies of EBNA2448–471 with Tfb1PH and CBP KIX. Histogram showing the variation in chemical shifts observed in the 1H-15N HSQC spectra of 15N-labeled EBNA2448–471 following the addition of either (A) Tfb1PH or (B) CBP KIX. Changes in chemical shifts are represented by Δδ = [(0.17ΔNH)2+(ΔHN)2]1/2, where ΔNH and ΔHN is the chemical shift difference between the two signals in ppm. (PDF) [file ppat.1004042.s003.pdf]

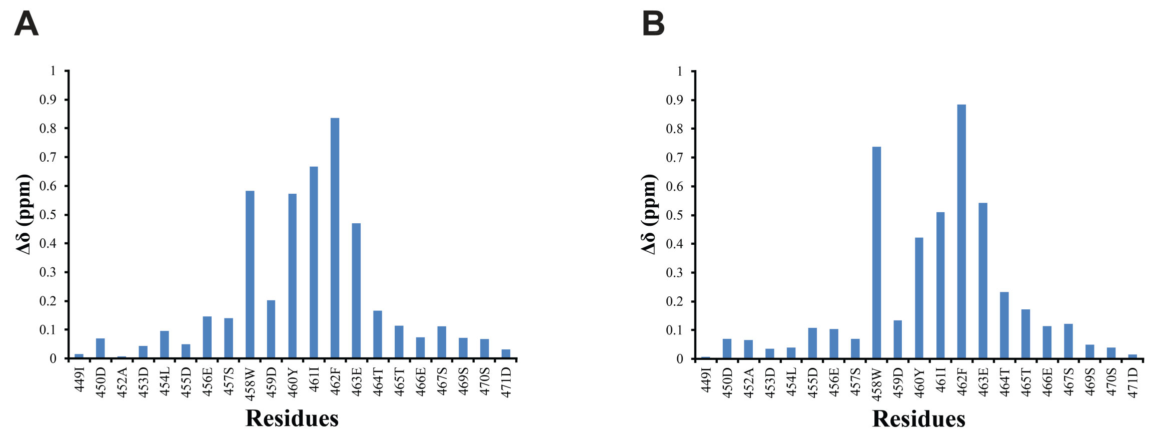

Supplementary Figure S3. NMR mapping studies of EBNA2$_{448\text{-}471}$ with Tfb1PH and CBP KIX. Histogram showing the variation in chemical shifts observed in the ^{1}H-^{15}N HSQC spectra of ^{15}N-labeled EBNA2$_{448\text{-}471}$ following the addition of either (**A**) Tfb1PH or (**B**) CBP KIX. Changes in chemical shifts are represented by $\Delta\delta = [(0.17\Delta N_H)^2 + (\Delta H_N)^2]^{1/2}$, where ΔN_H and ΔH_N is the chemical shift difference between the two signals in ppm.
