# Supplementary material for: Structural and Functional Characterization of a Complex between the Acidic Transactivation Domain of EBNA2 and the Tfb1/p62 Subunit of TFIIH
Source: PLoS Pathog. 2014 Mar 27;10(3):e1004042. doi: 10.1371/journal.ppat.1004042 (PMC3968163; doi:10.1371/journal.ppat.1004042)
Supplement: Figure S4 — Comparison of the TADs of p53, VP16 and EBNA2 in complex with Tfb1PH. The interface of the Tfb1PH in complex with the TAD of EBNA2, p53 and VP16, where Tfb1PH is shown as molecular surface (blue), In A–B, the TAD of EBNA2 is shown as a ribbon (orange) and the three key hydrophobic residues of the ΦXXΦΦ motif (W458, I461 and F462) of EBNA2 are shown in stick (orange). In A, the view is from the C-terminus of the EBNA2 helix. In B, the view is from the N-terminus of the EBNA2 helix. In C–D, the TAD p53 is shown as a ribbon (green) and the three key hydrophobic residues of the ΦXXΦΦ motif (I50, W53 and F54) of p53 are shown in stick (green). In C, the view is from the C-terminus of the p53 helix. In D, the view is from the N-terminus of the p53 helix. In E–F, the TAD VP16 is shown as a ribbon (magenta) and the three key hydrophobic residues of the ΦXXΦΦ motif (F475, M478 and F479) of VP16 are shown in stick (magenta). In E, the view is from the C-terminus of the VP16 helix. In F, the view is from the N-terminus of the VP16 helix. (PDF) [file ppat.1004042.s004.pdf]

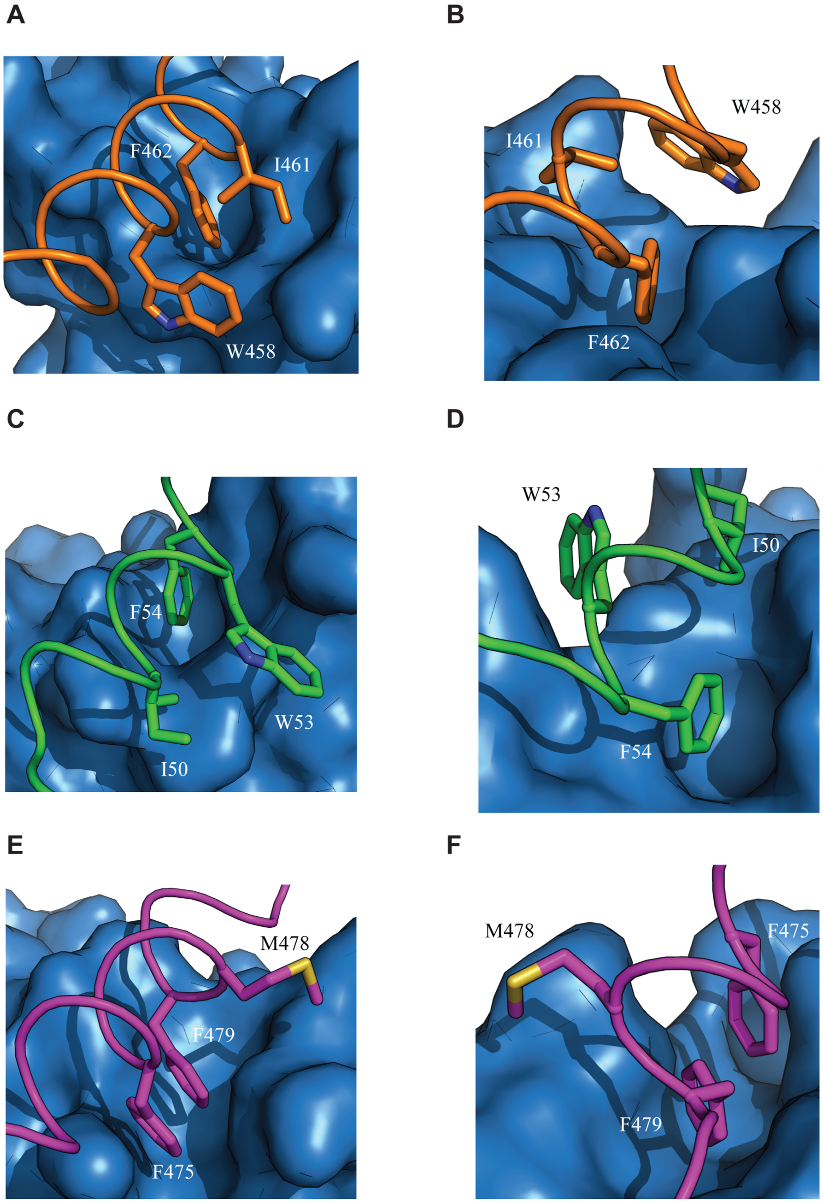

Supplementary Figure S4. Comparison of the TADs of p53, VP16 and EBNA2 in complex with Tfb1PH. The interface of the Tfb1PH in complex with the TAD of EBNA2, p53 and VP16, where Tfb1PH is shown as molecular surface (blue), In **A**-**B**, the TAD of EBNA2 is shown as a ribbon (orange) and the three key hydrophobic residues of the ΦXXΦΦ motif (W458, I461 and F462) of EBNA2 are shown in stick (orange). In **A**, the view is from the C-terminus of the EBNA2 helix. In **B**, the view is from the N-terminus of the EBNA2 helix. In **C**-**D,** the TAD p53 is shown as a ribbon (green) and the three key hydrophobic residues of the ΦXXΦΦ motif (I50, W53 and F54) of p53 are shown in stick (green). In **C**, the view is from the C-terminus of the p53 helix. In **D**, the view is from the N-terminus of the p53 helix. In **E-F,** the TAD VP16 is shown as a ribbon (magenta) and the three key hydrophobic residues of the ΦXXΦΦ motif (F475, M478 and F479) of VP16 are shown in stick (magenta). In **E**, the view is from the C-terminus of the VP16 helix. In **F**, the view is from the N-terminus of the VP16 helix.
